# Methodology to Forecast Volume and Cost of Cancer Drugs in Low- and Middle-Income Countries

**DOI:** 10.1200/JGO.17.00114

**Published:** 2018-02-13

**Authors:** Yehoda M. Martei, Sebathu Chiyapo, Surbhi Grover, Christina Hanna, Scott Dryden-Peterson, Malebogo Pusoentsi, Lawrence N. Shulman, Neo Tapela

**Affiliations:** **Yehoda M. Martei**, **Surbhi Grover**, **Christina Hanna** and **Lawrence N. Shulman**,University of Pennsylvania, Philadelphia, PA; **Sebathu Chiyapo**, Princess Marina Hospital; **Surbhi Grover**, Botswana-UPenn Partnership; **Scott Dryden-Peterson** and **Neo Tapela**, Botswana Harvard AIDS Institute Partnership; and **Malebogo Pusoentsi** and **Neo Tapela**, Ministry of Health and Wellness, Gaborone, Botswana; and **Scott Dryden-Peterson** and **Neo Tapela**, Brigham and Women’s Hospital, Division of Infectious Diseases, Boston, MA.

## Abstract

**Purpose:**

In low- and middle-income countries (LMICs), frequent outages of the stock of cancer drugs undermine cancer care delivery and are potentially fatal for patients with cancer. The aim of this study is to describe a methodologic approach to forecast chemotherapy volume and estimate cost that can be readily updated and applied in most LMICs.

**Methods:**

Prerequisite data for forecasting are population-based incidence data and cost estimates per unit of drug to be ordered. We used the supplementary guidelines from the WHO list of essential medicines for cancer to predict treatment plans and ordering patterns. We used de-identified aggregate data from the Botswana National Cancer Registry to estimate incident cases. The WHO Management Sciences for Health International Price Indicator was used to estimate unit costs per drug.

**Results:**

Chemotherapy volume required for incident cancer cases was estimated as the product of the standardized dose required to complete a full treatment regimen per patient, with a given cancer diagnosis and stage, multiplied by the total number of incident cancer cases with the respective diagnosis. The estimated chemotherapy costs to treat the 10 most common cancers in the public health care sector of Botswana is approximately 2.3 million US dollars. An estimated 66% of the budget is allocated to costs of rituximab and trastuzumab alone, which are used by approximately 10% of the cancer population.

**Conclusion:**

This method provides a reproducible approach to forecast chemotherapy volume and cost in LMICs. The chemotherapy volume and cost outputs of this methodology provide key stakeholders with valuable information that can guide budget estimation, resource allocation, and drug-price negotiations for cancer treatment. Ultimately, this will minimize drug shortages or outages and reduce potential loss of lives that result from an erratic drug supply.

## INTRODUCTION

The cancer burden is increasing in low- and middle- income countries (LMICs). In 2012, GLOBOCAN estimated that there were 14.1 million new cancer occurrences and 8.2 million cancer deaths worldwide. Fifty-seven percent of the new cancer occurrences and 65% of cancer deaths occurred in LMICs.^1^

The obstacles to cancer control in LMICs include poor infrastructure, inadequate access to essential cancer medicines and other cancer services, and lack of trained human resources.^2,3^ Even in places where adequate infrastructure and human personnel exist, there is a variable and often critical lack of access to chemotherapy and other systemic cancer therapies as well as to supportive medicines for cancer control. Although cost is a limiting factor for access to care, suboptimal forecasting of cancer drugs volumes also results in an inconsistent supply of drugs and frequent outages of stock (ie, stock outs). These outages compromise therapy for patients with cancer, especially for those who present with potentially curable diseases. Currently, 46 medicines are on the WHO Model List of Essential Medicines to treat many different types of cancer. Each cancer type requires different combinations and doses of these medicines, which makes forecasting a challenge for countries with limited resources, and which often results in stock outs.

Stock outs of essential systemic therapies can convert a potentially curative regimen to one that has no chance for cure, and stock outs result in excess toxicities and preventable loss of life. For a young patient with Hodgkin lymphoma, for example, use of four medicines (doxorubicin, bleomycin, vinblastine, and dacarbazine) results in an 85% cure rate. However, a stock out of doxorubicin for all or parts of the prescribed six cycles of therapy significantly reduces the cure rate, possibly to zero, which results in needless loss of life.

To meet the need for scale up of cancer programs in LMICs, countries must develop a forecasting methodology so that an adequate supply of needed pharmaceuticals can be procured to ensure that patients receive an uninterrupted supply of essential medicines and health products for cancer care. In most high-income countries (HICs) with complete real-time consumption data, forecasting is based on the method of exponential smoothing that predicts a value on the basis of the consumption for the prior period, with adjustment of error for the forecast, whereby recent data are weighted more heavily that older data.^4^ This method is not effective in LMICs, where complete consumption data often are not available and where prior demand or usage is confounded by nonuse of drug regimens caused by stock outs and misuse that results from lack of standardized guidelines. In addition, in many LMICs, restocking can take weeks to months and so may not meet the need of individual patients on timed systemic therapy schedules, in which delays in treatment can result in inferior outcomes.

The aim of this study is to describe a methodologic approach to forecast cancer drug volumes and cost estimation that can be easily updated and applied in most LMICs by using either national cancer registry data or GLOBOCAN estimates of cancer burden. The output of this process will provide national ministries of health, international partners, and various cancer control programs with estimates of cancer drug volumes for patients expected to be treated and relevant cost data for budget and resource allocation required for scaling-up cancer programs.

## METHODS

### Treatment Guidelines

Standardized treatment guidelines are needed to forecast cancer drug volumetric requirements. These guidelines may reflect an oncologist’s region of training: for example, National Comprehensive Cancer Network (NCCN) guidelines apply in the United States, and European Society for Medical Oncology (ESMO) guidelines apply in Europe. Data can be refined by using country-specific guidelines that account for additional regimens that are based on unique incidence patterns and availability of local resources. Regardless of which guideline is used, consistency in adherence to the treatment pathways is recommended to ensure predictable therapeutic plans for patients. For the purpose of this study, WHO supporting documents for the Essential Medicines List (EML) of 2015 were used to map therapeutic plans for individual cancer occurrences.^5^

### Patient Population

Cancer incidence and prevalence data as well as the proportion of diagnosed patients who access cancer care are critical data to estimate the need for cancer medicines. The more precise the data, the more accurate the drug forecasting, which results in an ideal balance that avoids both stock outs and overstock or expiration of drugs on shelves. National cancer registries and GLOBCAN data can be used to estimate initial cancer drug volumes and costs in countries with centralized national procurement protocols. Hospital register data also can be used for procurement at individual facilities in countries where medicine procurement is decentralized. The most accurate data will be derived from a national cancer registry that has high rates of case capture.

The application of the methodology was performed with data from Botswana, which is a middle-income country in sub-Saharan Africa with a population of 2.25 million.^6^ The gross domestic product (GDP) in the country is 15.27 billion US dollars; total health expenditure represents 5.4% of the GDP.^6^ The drug volumes in this study were estimated with data from the Botswana National Cancer Registry (BNCR). Data were limited to the 10 most commonly diagnosed cancers in men and women. The most recent update of the registry provides cumulative incidence over 8 years, from 2005 through 2012. Annual incidence was estimated as an average of diagnoses that occurred during the 8-year time period.

### Stage, Tumor Characteristics, and Survival Data

Cancer stage and other tumor characteristics determine what therapy is prescribed for patients. Data on stage distribution should be extracted from tumor registry data, when available, or extrapolated from publications and abstracts within the region. Additional information on distribution of breast cancer molecular subtypes, or on mutation status for cancers such as lung cancer and melanoma, are cancer specific and vital to prescribe and forecast the need for the respective targeted therapy in countries where these therapies are included in the national essential medicines list. Likewise, these data can be estimated initially with published data or abstracts from the region.

For patients with curable disease, duration of treatment is finite and can be directly incorporated into the formula. For patients with metastatic disease, the duration of treatment is until tumor progression or death. Unless otherwise specified, time to progression (TTP) was estimated as 6 months for any given regimen used in the treatment of all stage IV solid tumors. The volumes of targeted therapy for hormone receptor–positive and human epidermal growth factor receptor 2 (HER2)–positive metastatic breast cancer were estimated with a treatment duration period of 1 year.

Incomplete stage data as well as molecular phenotypes of breast cancer are captured in the BNCR. Stage distribution was estimated from the Botswana Prospective Cancer Cohort, a long-term cohort study by the Botswana Harvard AIDS Institute Partnership on patients with newly diagnosed cancer in Botswana who present for oncologic treatment, and receptor status was estimated from pathology review data from the National Health Laboratory.^7^

### Cost of Cancer Drugs

Cost per unit data are available publicly on the WHO Management Sciences for Health International Drug Price Indicator Guide.^8^ This can be used for budget projections, with the knowledge that pharmaceutical pricing is not transparent and varies widely. In countries where set prices with specific suppliers have been negotiated previously, those price estimates can be used instead. A comparison of set price and median market prices on the world market also is recommended to make budget estimations. We used the Management Sciences for Health median price per unit vial or pack size per treatment regimen per patient to estimate total cost of cancer drugs for the population.

### Assumptions

Dosages of cancer medicines are prescribed in most cases on the basis of body surface area (BSA), weight, or—in a few cases—creatinine clearance. Estimates of 1.8m^2^ for BSA, 62 kg for average weight, and 100 mL/min by the Cockcroft-Gault formula for creatinine clearance were used. These default estimates can be replaced by country-specific weight and BSA trends. Regimens with dose ranges were estimated by using the upper range. The net result is likely to be a slight overestimation of the requirements for the population.

### Ethical Review

This study was exempted from the requirement of institutional review board review by the Botswana Health Research and Development Committee.

## RESULTS

### Patient Population

Data from the BNCR were used to estimate the annual incidence of the most common cancers diagnosed in Botswana. Table 1 lists the combined incidence of the top 10 cancers diagnosed in men and women in Botswana compared with GLOBOCAN estimates. Anatomic diagnoses with no pathologic correlates, such as eye and skin, other, were excluded. Classifications of mouth and trachea were excluded as nonspecific diagnoses to which a particular treatment could not be applied. Although liver cancer is the number eight and 10 most commonly diagnosed cancer in men and women in Botswana, respectively, we did not include this in the analyses because of the lack of efficacious therapy.

**Table 1:**
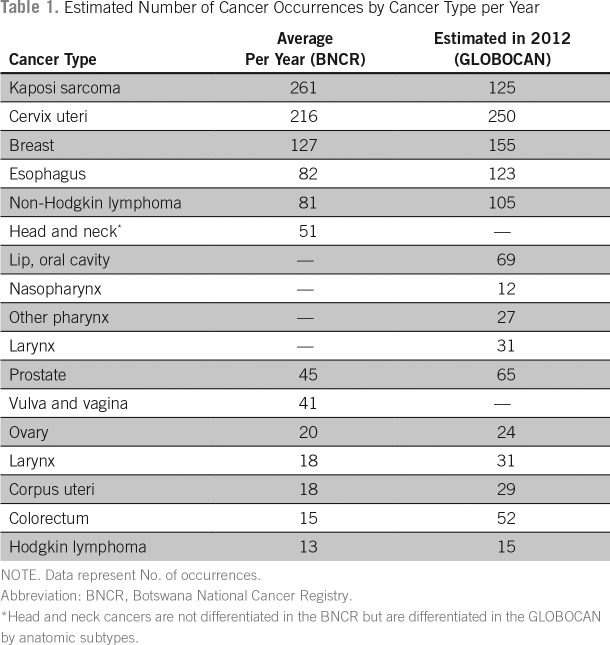
Estimated Number of Cancer Occurrences by Cancer Type per Year

**Fig 1:**
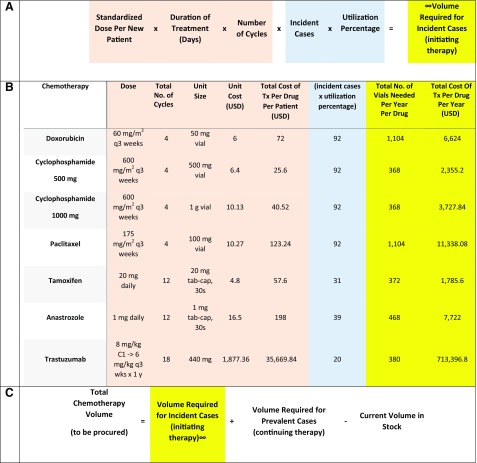
Row 1A describes formula for estimating chemotherapy volume to be procured. The rows in 1B illustrate the chemotherapy forecasting formula applied to all patients with local advanced breast cancer. Row 1C shows chemotherapy volume forecasts accounting for prevalent cases.

The volume of chemotherapy and other cancer medicines required for a specific disease was calculated first by computing the volume required per patient (Figure 1A orange-shaded area). This used the standardized dose per new patient specific to the cancer type. The total dose per cycle then was calculated on the basis of the number of days of therapy needed per cycle (eg, days 1 and 8 for most gemcitabine doses). Total therapy course was calculated by multiplying total dose per cycle (eg, number of vials or tablets) by the total number of cycles. In the blue-shaded areas of Figure 1A formula, the individual patient estimates are multiplied by the total population that needs the drug. In breast cancer, as illustrated in the text example in the following paragraph, the percent of utilization estimates take into account which fraction of patients will require a given drug. For most drug regimens, this fraction is 100%; however, for targeted therapy, this number is less than 100%, as demonstrated in the example for HER2-positive breast cancer (Fig 1B formula).

Figure 1B formula illustrates how formula A was applied to occurrences of locally advanced nonmetastatic breast cancer cases, for which there are 92 occurrences per year. The defined regimen contains standard doses used in the clinic setting and the number of finite cycles recommended in the neoadjuvant or preoperative setting. A utilization rate of 100% was estimated for doxorubicin, cyclophosphamide, and paclitaxel (AC/T), which serves as a backbone regimen for all locally advanced disease regardless of receptor subtype. Utilization for tamoxifen was based on 75% hormone receptor–positive status^7^ and used prior registry data to assign 45% of patients to a premenopausal status (ie, age younger than 50 years) at the time of diagnosis. The product of incident cases and utilization percentage for tamoxifen therefore, was approximately 31 (ie, 92 × 75% × 45%). HER2 positivity for the population was an estimated 21.6% (Fig 1B).^7^

For practical purposes, when cancer medicines are procured, it is important to realize that the estimates used here represent incident cases only. When available, the volume for prevalent cases should be estimated and added when relevant (eg, in patients who are receiving their second or third year of tamoxifen for hormone receptor–positive breast cancer). To avoid overstock, the final estimates should take into account current stock before an order is placed. The formula is illustrated in Figure 1C. The current volume in stock will be either a discrete number or zero if the drug is out of stock.

### Cost of Cancer Drugs

**Fig 2:**
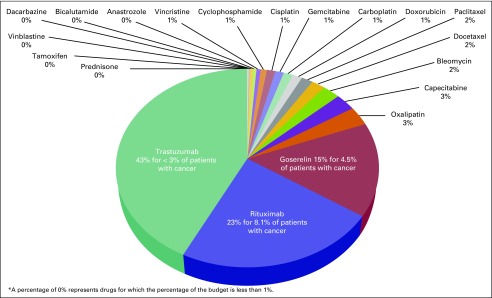
Proportional distribution of chemotherapy costs (projected chemotherapy budget per year is 2.3 million US dollars): percentage of budget by specific drug.

The estimated costs of cancer drugs needed to treat the 10 most common cancers diagnosed among men and women in the public sector in Botswana was approximately 2.3 million US dollars per year. These costs are limited to pharmaceuticals only and do not include other costs associated with cancer care and delivery of cancer drugs. An estimated 66% of the budget was attributed to the costs of rituximab and trastuzumab used by approximately 10% of the cancer population. Generic cancer drugs cost significantly less and cover most of the cancer population. Table 2 lists an aggregate cost of cancer drugs; Table 3 lists aggregate forecasted volumes; and Figure 2 shows the proportional costs of individual pharmaceuticals and proportion of cancer patients covered by the specified agent.

**Table 2:**
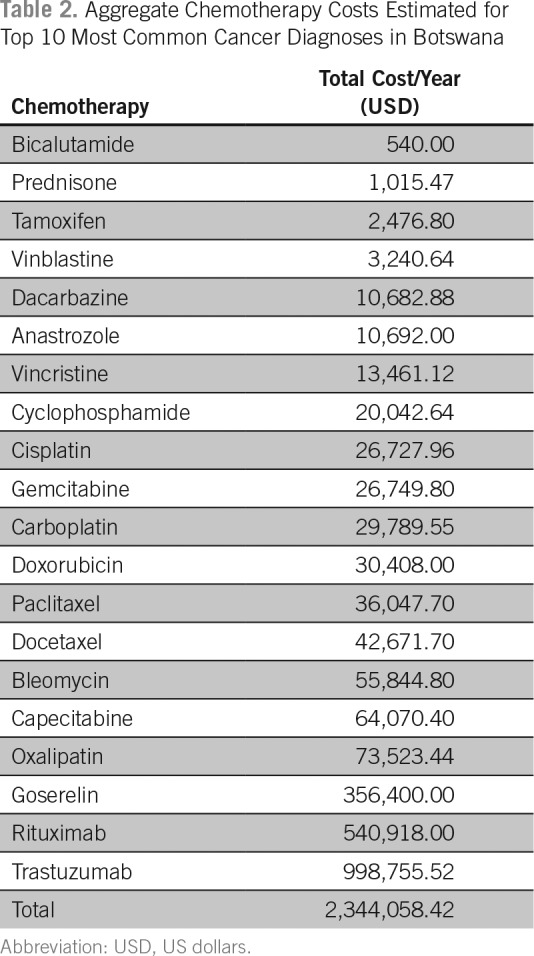
Aggregate Chemotherapy Costs Estimated for Top 10 Most Common Cancer Diagnoses in Botswana

**Table 3:**
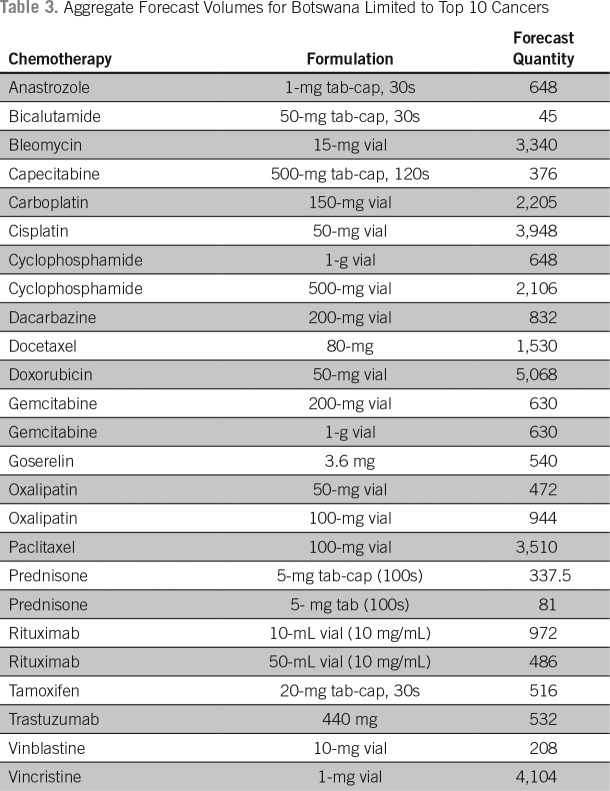
Aggregate Forecast Volumes for Botswana Limited to Top 10 Cancers

## DISCUSSION

We described in this study a systematic approach to estimate cancer drug volumes in LMICs; the approach is reproducible and can be applied easily in countries with varying levels of cancer registry data, including minimal data on cancer incidence, or with varying quality of national registry databases. The output generated by this process provides key quantifications for cancer drug volumes and cost data that can be shared with stakeholders.

To our knowledge, these results provide the first aggregate volumetric estimates of cancer drugs and total budget needs for a given year in Botswana. Our analysis showed critical insights into the distributional costs of cancer drugs that were not previously appreciated. We showed that, despite the overwhelming efficacy data that led to the inclusion of trastuzumab and rituximab in the updated version of the WHO Essential Medicines List and also the Botswana National Essential Medicines List,^5^ the costs of these relatively newer therapies account for an extraordinarily large component of the annual budget for cancer drugs. Specifically, although trastuzumab is used by only 3% of the patient population (those with HER2-positive breast cancer), it accounts for more than 40% of the budget for cancer drugs. These estimates provide key data that can be used by the Ministry of Health to formulate criteria and treatment pathways for which patients should be covered by medicines included on the National Essential Medicines List. The data may lead stakeholders to limit costly therapy to patients being treated with curative intent only or may cause stakeholders to choose not to purchase these drugs at all. The cost estimates also provide supportive data about potential cost barriers to access, which can be leveraged by stakeholders to negotiate lower drug prices or implement policies to increase access and drive down costs. In a recent press release, the WHO announced one such an initiative: an affordable drug pilot program to increase access to more affordable biosimilars for trastuzumab and rituximab in LMICs.^9^

This approach involved several assumptions, the net result of which is an overestimation of cancer drug volumes, because not all patients with incident cancers seek or have access to care. However, the process of volumetric forecasting is an iterative process that continuously takes into account current stocks at the beginning of each procurement cycle. This methodology ensures more precise estimation with each iteration of the procurement process and more complete data collection. Ideally, a procurement cycle that happens biannually will ensure that adjustments are made continuously to avoid stock outs and also will minimize the likelihood of overstock and pharmaceutical expiration in storage. Although this formula incorporates incident and prevalent cases, we did not calculate the prevalent cases specifically for Botswana because of the lack of complete data. We do realize that these patients have to be accounted for in final procurement estimates. For instance, although the incidence of chronic myeloid leukemia is extremely low, the prevalence of the disease is relatively high because of highly effective treatment with imatinib, which needs to be taken indefinitely, and high rates of adherence among patients in Botswana. Currently, 40 patients with chronic myeloid leukemia receive imatinib, and the cost is approximately 3,000 US dollars per month of treatment per patient.

Botswana has a population of 2.25 million people and therefore faces unique challenges related to its relatively low absolute numbers of cancer occurrences. This translates into low volumes of cancer medicines that may not meet the minimum order requirement for competitive suppliers. Aggregation of forecasting data for the year or consolidation of regional procurements are potential ways in which Botswana can meet minimum order requirements and subsequently negotiate lower cancer drug prices for patients with cancer. The output data from this methodology are being used in the current procurement cycle to determine quantities of cancer medicines to be purchased through the public sector in Botswana. The data also have generated ongoing discussions about the cost of trastuzumab and the financial costs on the public health sector versus the potential benefits gained by offering this treatment only to patients in the curative setting or to all patients with breast cancer regardless of disease stage.

In LMICs where quality registry data are scant, this method can be easily applied by using GLOBOCAN data as a critical first step to map out a national cancer control plan. One must also consider a country’s capacity to treat patients with cancer, because accessibility of quality cancer care will determine how many patients can be treated in a given year. The public-sector coverage of cancer incidence will differ between countries on the basis of incidence patterns, gross domestic product, and total health care expenditure in the public and private sector. Specifics must be determined for each country on the basis of cancer incidence profiles and economic considerations. Regardless, this methodology will go a long way toward minimization of stock outs and prevention of unnecessary deaths caused by an erratic supply of cancer drugs. It also starts to provide critical data to illustrate how expensive prices of cancer medicines may price out the world’s poorest from receiving potentially life-saving drugs. These data can be used to negotiate lower prices for regions of the world that cannot afford to spend an exorbitant 40% of its national budget on cancer drugs for 3% of the population—a population, nonetheless, that will derive significant cure rates and life-prolonging benefits.^10^ As mentioned earlier, these output data are essential for all stakeholders to begin to formulate a cancer care plan in LMICs, with the goal of reducing the current mortality gap between high-income countries and LMICs.

## References

[1] Ferlay J, Soerjomataram I, Dikshit R (2015). Cancer incidence and mortality worldwide: Sources, methods and major patterns in GLOBOCAN 2012. Int J Cancer.

[2] Farmer P, Frenk J, Knaul FM (2010). Expansion of cancer care and control in countries of low and middle income: A call to action. Lancet.

[3] Gelband H, Sankaranarayanan R, Gauvreau CL (2016). Costs, affordability, and feasibility of an essential package of cancer control interventions in low-income and middle-income countries: Key messages from Disease Control Priorities, third edition. Lancet.

[4] D’Sa MM, Nakagawa RS, Hill DS (1994). Exponential smoothing method for forecasting drug expenditures. Am J Hosp Pharm.

[5] Shulman LN, Wagner CM, Barr R (2016). Proposing essential medicines to treat cancer: Methodologies, processes, and outcomes. J Clin Oncol.

[6] The World Bank https://data.worldbank.org/country/botswana.

[7] Narasimhamurthy M, Kayembe MK, Ambarishan M Immunohistochemical profile of breast cancer patients in Botswana.

[8] Management Sciences for Health: International Drug Price Indicator Guide, 2014. http://mshpriceguide.org/wp-content/uploads/2016/06/MSH-International-Drug-Price-Indicator-Guide-2014.pdf

[9] WHO: News, May 4, 2017: WHO to begin pilot prequalification of biosimilars for cancer treatment. http://www.who.int/mediacentre/news/releases/2017/pilot-prequalification-biosimilars/en/

[10] Piccart-Gebhart MJ, Procter M, Leyland-Jones B (2005). Trastuzumab after adjuvant chemotherapy in HER2-positive breast cancer. N Engl J Med.

